# Acute Gastric Necrosis Induced by Caustic Substance Ingestion: A Case Report

**DOI:** 10.7759/cureus.74719

**Published:** 2024-11-29

**Authors:** Wala O Sati, Mohammad Abdow, Doaa M Sabir, Hadeel Elhassan, Waleed Salem

**Affiliations:** 1 Emergency Department, Hamad Medical Corporation, Doha, QAT; 2 Emergency Medicine, Hamad Medical Corporation, Doha, QAT

**Keywords:** caustic acid ingestion, caustic ingestion injury, corrosive ingestion, gastric mucosal necrosis, hydrochloric acid

## Abstract

Ingestion of caustic substances is a common toxicological emergency, often resulting in significant morbidity and mortality. Accidental ingestion of these toxic agents is most prevalent among children, who may encounter household cleaners and other hazardous substances out of curiosity. In contrast, adults often ingest caustic agents in the context of self-harm or suicide attempts. These agents can cause extensive damage to the gastrointestinal tract, leading to serious complications, including perforation, strictures, and systemic toxicity. Both acids and alkalis are particularly dangerous, as they can create irreversible injuries depending on their concentration and duration of exposure.

In this report, we detail the case of a middle-aged man who accidentally ingested a corrosive cleaner containing hydrochloric acid. One day post-ingestion, he presented with throat pain and odynophagia, raising immediate concern for possible gastrointestinal injury. An upper gastrointestinal endoscopy was performed, which revealed findings indicative of gastric necrosis, confirming the severity of the injury. Unfortunately, the patient lost follow-up after being discharged from the hospital.

This case underscores the critical importance of recognizing the type of caustic substance involved in such emergencies and highlights the need for prompt medical intervention. Immediate identification of the ingested material, combined with rapid treatment, is essential to mitigate damage and improve patient outcomes.

## Introduction

Accidental and intentional caustic ingestions present significant challenges in clinical practice. Children are particularly at risk of accidental exposure to household products, while adult cases tend to be more severe due to suicidal intent, with mortality rates ranging from 10% to 20% and potentially rising to 78% in cases of intentional ingestion. Caustic substances have the potential for severe morbidity and mortality, primarily affecting the upper digestive tract. The severity and location of gastrointestinal (GI) injury from corrosive substances depend on factors like product formulation, concentration, contact duration, and whether food is present in the stomach. 

Caustic acids and alkalis, prevalent in both domestic and industrial settings, can lead to extensive tissue damage upon ingestion or skin contact, necessitating immediate recognition and therapeutic interventions. The range of injuries can vary from mild mucositis to severe necrosis. Caustic ingestions, especially those involving strong acids or bases, can cause severe tissue damage to the gastrointestinal tract. Alkalis, such as sodium hydroxide, lead to liquefactive necrosis, dissolving tissues deeply, often causing severe GI damage, while acids like sulfuric acid cause coagulative necrosis, forming a protective layer that limits penetration, thus commonly affecting the stomach and duodenum. Injuries progress through four stages, from initial necrosis with healing occurring between five and 14 days and risks of perforation and stricture formation thereafter to potential scarring and stricture formation. However, both can inflict severe, full-thickness injuries. The severity of damage depends on factors like the substance's pH, physical state, contact time, quantity, and concentration. Volatile agents containing acids and bases can be particularly damaging as they disrupt protein metabolism, leading to coagulation, fluid loss, and compromised cell integrity [1].

Acidic substances such as hydrochloric acid, commonly found in household cleaners, are well known for causing gastrointestinal (GI) upset, particularly in the stomach, which is already exposed to a highly acidic environment. This can manifest as symptoms such as pain, vomiting, and dysphagia. If these signs and symptoms are not recognized and treated promptly, they can progress to severe complications, including GI perforation and peritonitis. Predicting GI injury based on symptoms is difficult, although dysphagia and vomiting may indicate significant GI tract lesions. 

Identification of the ingested substance and the immediate initiation of appropriate treatment and evaluation are crucial. Prompt endoscopy (or CT in some cases) assesses injury severity, and emerging risk-assessment tools aid in predicting the extent of damage. Timely treatment of caustic GI injuries is important for several reasons. It minimizes tissue damage by limiting the extent of necrosis and preserving surrounding healthy tissues, and decreasing the risk of complications such as perforation. Additionally, it helps in preventing secondary infections that can arise from damaged and necrotic tissue, which can worsen the patient’s condition. Immediate interventions include intravenous (IV) fluids, proton pump inhibitors (PPIs), H2 antagonists, and pain management to promote healing. Surgical intervention is required for perforations or severe strictures, with minimally invasive techniques preferred. Prevention of strictures may involve corticosteroids, though their effectiveness is debated. Other treatments, such as stents or intralesional agents, are under investigation. 

The prognosis depends on the injury's severity, with complications like strictures and cancers as long-term concerns. Ultimately, early intervention can significantly improve patient outcomes and reduce the high morbidity and mortality rates associated with caustic ingestion cases. Healthcare professionals must be vigilant in monitoring patients for signs of deterioration, ensuring that follow-up care addresses both acute injuries and any potential long-term complications [2].

## Case presentation

This case involves a 40-year-old male with no significant medical history who presented to the emergency department (ED) after accidentally ingesting approximately 10-20 ml of an acidic toilet cleaner containing an unknown concentration of hydrochloric acid. The incident occurred one day prior to his presentation, and the patient experienced four episodes of non-bloody emesis on the same day of the event. When he attended ED, he was complaining of throat pain and irritation while swallowing food. He denied any abdominal pain, diarrhea, respiratory distress or cough, chest pain, or any other complaints, and he denied suicidal thoughts.

**Figure 1:**
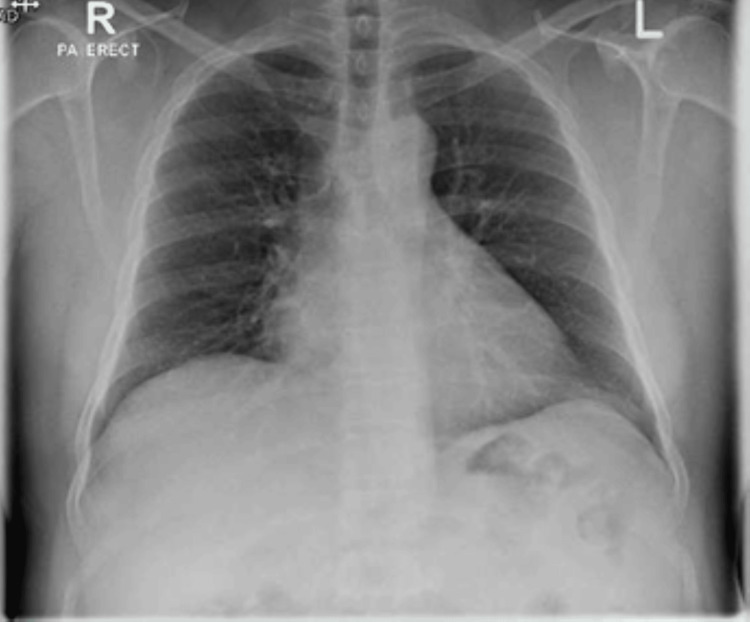
Normal chest X-ray. PA: posterior to anterior.

Upon examination in the ED, the patient was alert and conscious, with normal bilateral breath sounds and clear chest auscultation. His heart sounds were also normal. A throat examination revealed mild congestion and redness. Vital signs were stable: SpO_2_ 98% on room air, respiratory rate 18 breaths per minute, heart rate 70 bpm, blood pressure 130/82 mmHg, and temperature 36.6°C. His venous blood gas (VBG) results are shown in Table 1. His complete blood count (CBC) and biochemistry reports were unremarkable, and a chest X-ray was normal (Figure 1).

**Table 1:** Blood gas results. BE: base excess.

Parameter	Result	Reference range
PH	7.32	7.320-7.420
PCO_2_	58 mmHg	45-55
PO_2_	20 mmHg	25-40
Na	141 mmol/L	135-145
K	3.7 mmol/L	3.5-5.0
Cl	103 mmol/L	96-110
HCO_3_	30.2 mmol/L	23.0-29.0
Lactate	0.90 mmol/L	0.50-2.20
BE	2.2 mmol/L	2.0-2.0

At this stage, toxicology specialists were consulted, and they recommended an evaluation by an ENT specialist for assessment of the patient's airway, as well as a GI consultation for potential endoscopy, given the working diagnosis of corrosive ingestion injury. The ENT specialist conducted a fiberoptic examination, which showed a clear nasopharynx, oropharynx, and hypopharynx. The vocal folds were intact and mobile, and the epiglottis, pyriform fossa, and vallecula appeared normal.

**Figure 2:**
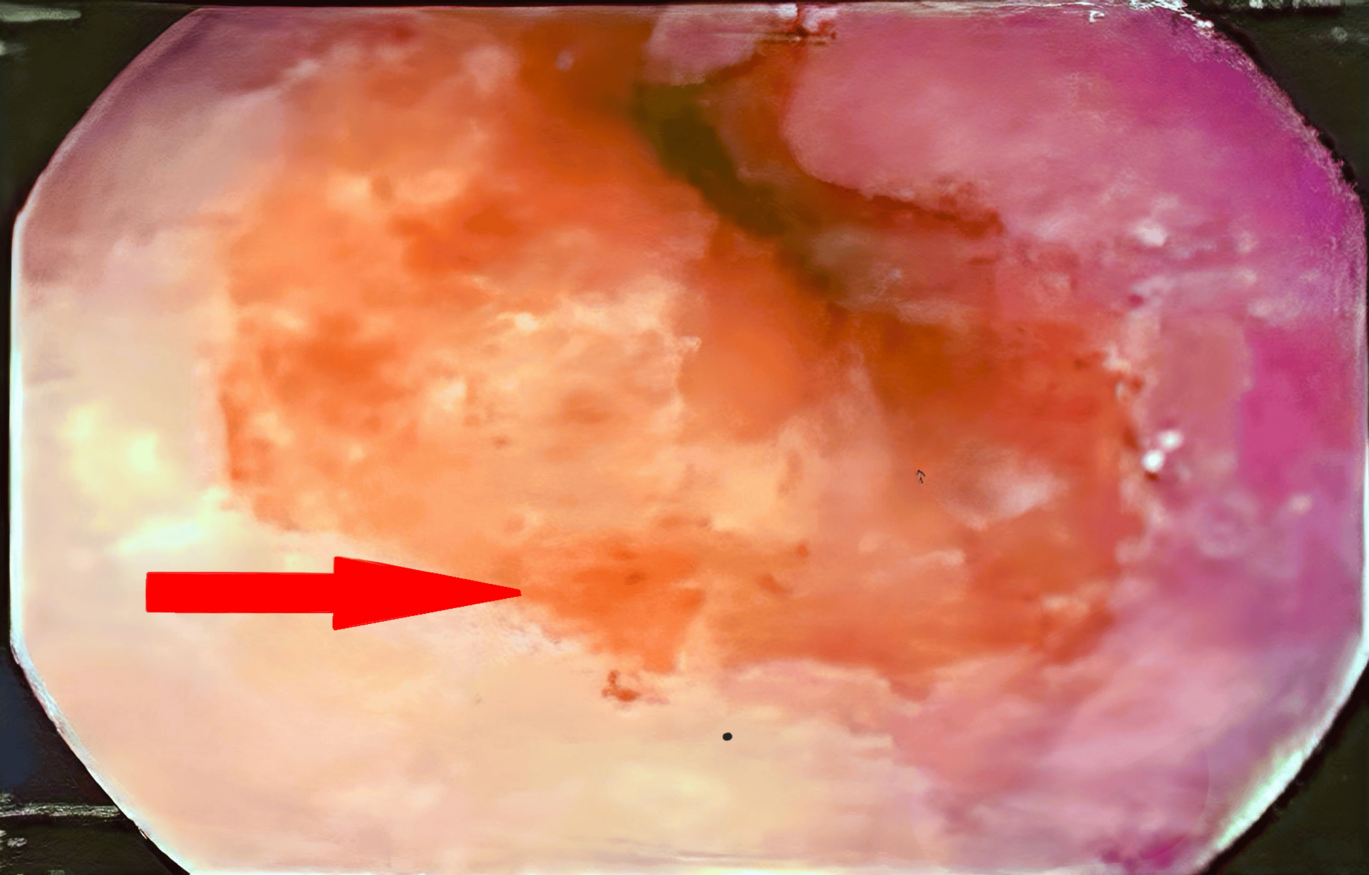
Endoscopic picture showing erythema in the lower esophageal mucosa.

**Figure 3:**
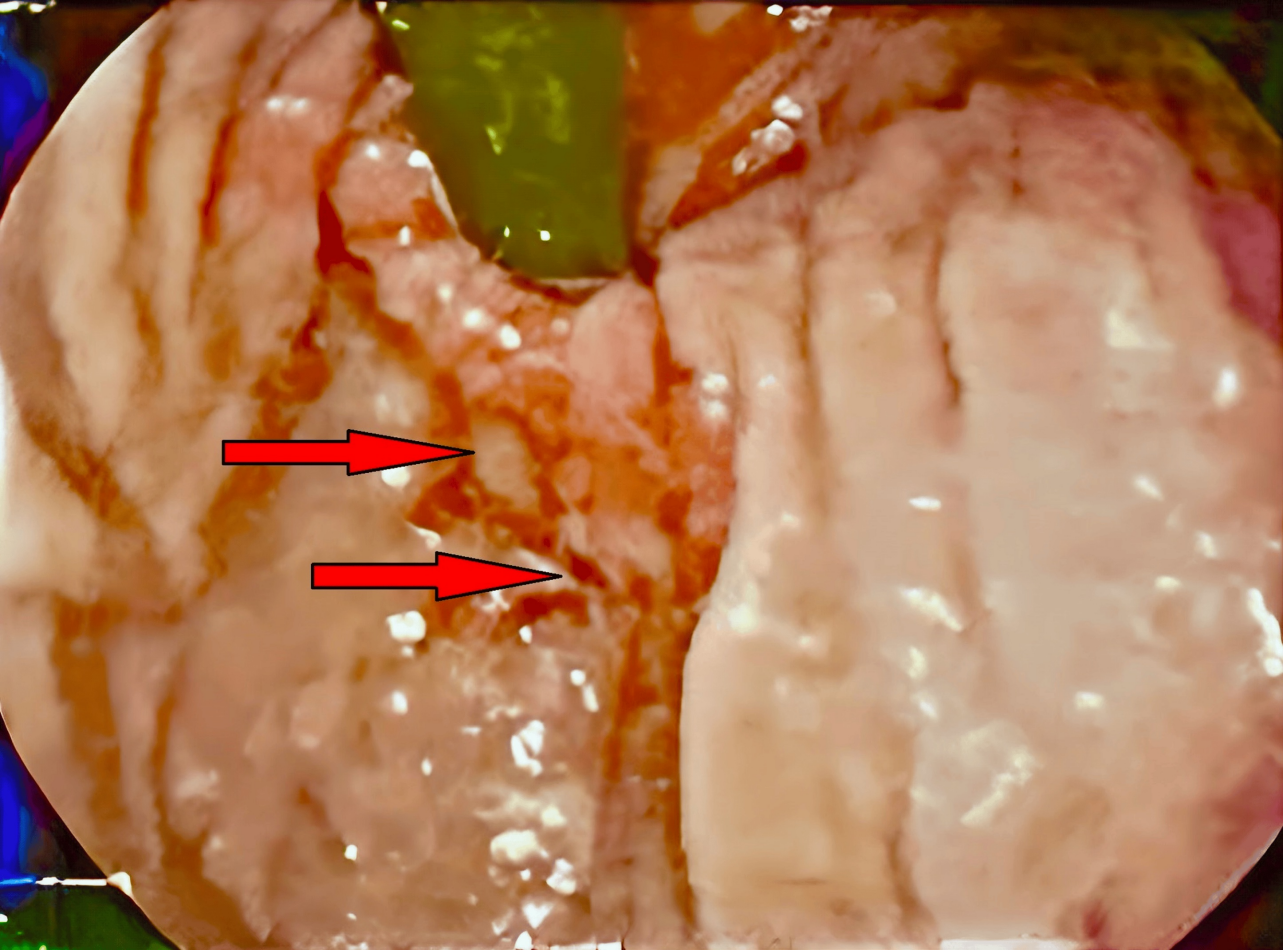
Endoscopic picture showing bleeding at the lesser curvature.

**Figure 4:**
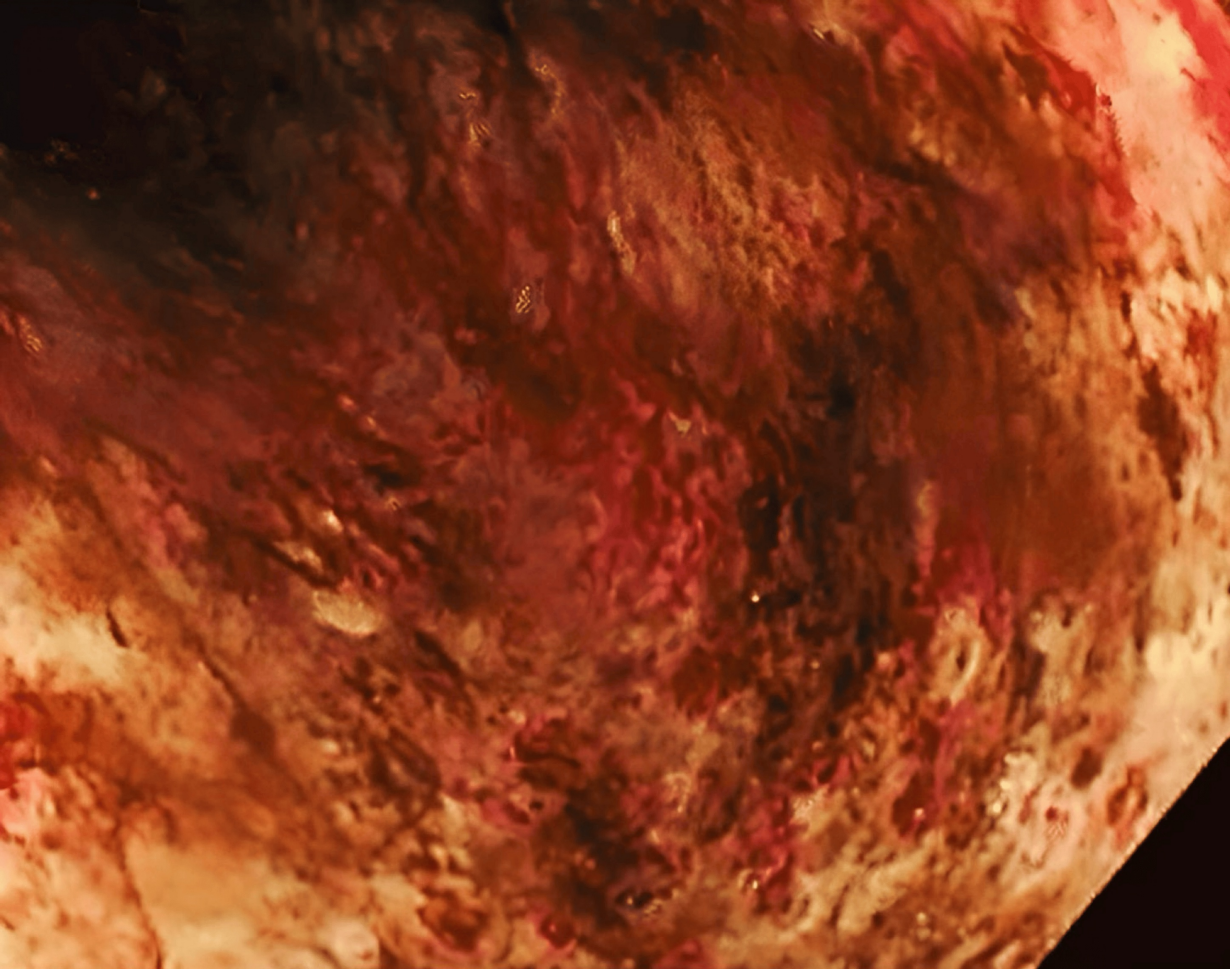
Endoscopic picture of the distal lesser curvature body showing black necrotic mucosa accompanied by erythema and bleeding.

An upper GI endoscopy was performed on the same day and revealed erythema in the epiglottis, lower esophageal mucosa, and antrum. The distal body and lesser curvature showed black necrotic mucosa accompanied by erythema and spontaneous bleeding. Additionally, erythema was observed in the duodenum (D1). In contrast, the mucosa of the second part of the duodenum (D2) appeared normal, which correlates with the endoscopic diagnosis of caustic ingestion injury-ZARGAR 3A (see Figures 2-4).

The GI team recommended that the patient remain fasting for 48 hours, followed by the introduction of a clear liquid diet, which was tolerated well for two days by the patient, followed by slowly escalating to a normal diet. A CT scan of the abdomen was recommended, and it was advised to monitor the patient in the hospital for at least seven days. In addition, continuing IV proton pump inhibitors (PPIs) twice daily and transitioning to oral tablets upon discharge was advised, and a follow-up endoscopy was scheduled in two months.

A CT scan of the abdomen was performed four days later, as the patient remained asymptomatic and showed no urgent signs, revealing no evidence of visceral perforation or peritonitis. The patient remained clinically stable throughout his hospital stay and was discharged home six days later in good condition. Unfortunately, he lost a follow-up after leaving the hospital.

## Discussion

According to the American Association of Poison Control Centers (AAPCC), over 200,000 people in the United States have been exposed to household cleaning products since 2000. Ingestion of alkaline or acidic substances is a well-documented cause of severe gastrointestinal (GI) tract damage. These corrosive agents can lead to immediate complications during their transit through the GI tract, including laryngospasms, perforations, necrosis, and mediastinitis, often resulting in fatal outcomes within hours. For patients who survive the initial acute phase, long-term complications may arise, such as esophageal strictures, gastric outlet obstructions, and even the development of squamous cell carcinoma or adenocarcinoma of the esophagus, typically occurring four to six weeks after the incident. GI injuries vary significantly, ranging from mild erythema to full-thickness necrosis. The literature extensively discusses the classification and management of these injuries, emphasizing the critical role of early endoscopic evaluation within 24 to 48 hours post-ingestion, as illustrated in this case. Early endoscopic findings can effectively categorize the severity of the injury and inform appropriate management strategies [3]. Research shows that even short exposure (10 seconds) to solutions can lead to extensive necrosis of the esophageal mucosa and deeper tissues, with more concentrated solutions causing complete wall destruction and injury to surrounding organs. Studies in animals reveal that liquid caustics lead to significant burns and inflammatory responses, often resulting in strictures, particularly with liquid caustics compared to granular forms. Overall, liquid corrosives pose a greater risk of serious long-term damage than solid forms [4].

In a case report involving a two-year-old girl, the authors detail severe complications following caustic ingestion, which led to significant gastrointestinal and respiratory damage. She presented with solid dysphagia and signs of malnutrition after accidentally ingesting 100 mL of NaOH 50 days prior. The patient spent 40 days in a local hospital receiving limited treatment, during which extensive tissue necrosis was identified, and imaging revealed severe esophageal stenosis. Dilation was performed using Savary dilators; however, the treatment failed after three months. During this time, a gastrostomy was placed to provide feeding, but esophageal replacement became unavoidable. Despite this, the parents declined further surgical intervention, and the patient was subsequently discharged. Three months later, she returned with respiratory distress and signs of malnutrition. Imaging showed atelectasis in the left lung and a tracheoesophageal fistula. The left lung was removed due to non-functionality. An attempt was made to isolate and remove the esophagus; however, due to significant adhesions and inflammation, only the distal portion could be isolated, with no fistula identified. Following an appendectomy, a colon conduit was created for replacement. This case highlights the potential severity of such incidents and underscores the need for comprehensive care strategies [5].

In another case, the author describes a patient who ingested 100 mL of 24% hydrochloric acid in a suicide attempt, which resulted in extensive gastric mucosal necrosis identified by esophagogastroduodenoscopy. A diagnostic laparoscopy confirmed transmural necrosis, necessitating a discontinuous laparoscopic gastrectomy. Remarkably, continuity was restored by the sixth postoperative day through a Roux-en-Y oesophagojejunostomy. The patient was discharged in good condition [6]. These cases describe that acids like hydrochloric acid are more common to cause more severe gastric than esophageal damage, a pattern observed in our case.

A case of 84-year-old man who ingested 150 mL of 7% hydrochloric acid in a suicide attempt developed extensive gastric necrosis and perforation in a follow-up contrast-enhanced CT, prompting surgical intervention. Laparotomy revealed almost complete gastric necrosis and pancreatic complications, leading to a subtotal gastrectomy with Billroth I anastomosis and pancreatosplenectomy. This case emphasizes that contrast-enhanced CT is superior to endoscopy for detecting digestive tract injuries shortly after caustic ingestion even small quantities of strong acids can lead to extensive necrosis, reinforcing the potential severity of our patient’s condition despite ingesting as small amount as 10-20 ml and highlights the importance of repeated imaging if initial results are normal such as the CT report in our case in the study [7].

Ingestion of alkaline substances typically causes more damage to the esophagus than to the stomach or duodenum, while acidic substances primarily injure the stomach. However, recent reports challenge this view, showing that extensive esophageal injuries or perforations can occur after acid ingestion. Both acid and alkaline substances can also harm the larynx, trachea, and bronchi. Alkaline ingestion leads to liquefaction necrosis, rapidly extending damage beyond the esophagus to the mediastinal wall until neutralized by tissue fluids. The prolonged presence of alkaline fluids worsens injuries, and while gastric acid can partially neutralize these substances, severe complications such as perforation and mediastinitis may arise. Liquefaction necrosis can last for several days, resulting in intravascular thrombus, mucosal inflammation, and ulceration, with chronic complications like stricture formation, depending on the depth of injury.

In contrast, acidic substances generally cause superficial coagulation necrosis, leading to tissue scar formation without deep injuries. Acids often cause immediate oropharyngeal pain, resulting in smaller volumes being ingested compared to alkaline. As acidic substances move quickly through the esophagus to the stomach, they predominantly damage the gastric antrum, especially if no food is present to help neutralize them. Studies suggested that worse outcomes have been reported with acid ingestion despite alkaline injuries being more severe, including higher rates of complications and ICU admissions. Some reports suggest that asymptomatic children who ingest low-potency caustic substances may not need endoscopy, while adults who intentionally ingest potent caustic substances should undergo emergency endoscopy, ideally it should be performed within 24 hours but safely up to 96 hours after ingestion, to assess potential esophageal or gastric injuries. Although it is contraindicated in hemodynamically unstable patients or those showing signs of perforation, respiratory distress, or severe edema, it helps anticipate prognosis and establish treatment plans [8].

Endoscopic assessment revealing black necrotic ulcers may suggest full-thickness injury. To enhance prognostic accuracy, grade 2 burns can be further classified into two subcategories: grade 2a, characterized by superficial ulcers, and grade 2b, involving deep ulcers. The latter is associated with a higher risk of stricture formation. In contrast to patients with limited necrosis (grade 3a), who have lower risks of complication and mortality, those with extensive necrosis (grade 3b) face remarkable risks, requiring surgical intervention. This new classification system aims to enhance the management of caustic injuries, as current treatment approaches remain controversial. Early surgical intervention may not be required for grade 3a injuries, whereas grade 3b injuries could benefit from prompt surgery to reduce complications [9]. This was similar to the patient described in this case report, who was diagnosed with ZARGAR 3A through endoscopy, despite a CT scan showing no further complications.

Management of acute corrosive ingestion focuses on initial resuscitation, assessing injury severity, treating early complications, maintaining nutrition, and preventing strictures. Initial resuscitation key principles include maintaining airway and circulation; tracheostomy and mechanical ventilation if necessary in case of laryngeal injuries. Meanwhile, supportive care is prioritized over specific antidotes. Although the efficacy of intravenous (IV) proton pump inhibitors (PPIs) is not established, they may be administered to patients with higher-grade injuries to protect the gastric mucosa and mitigate gastroesophageal reflux. Although the use of systemic steroids for corrosive esophagitis is controversial, some studies advised high-dose steroids as they may help prevent stricture formation. Routine broad-spectrum antibiotics are generally not recommended except for high-grade injuries and those on systemic steroids. Endoluminal dilatation is the preferred treatment for strictures in children, typically starting three to six weeks post-injury. If strictures are refractory to dilatation attempts, esophageal replacement surgery may be needed [10,11]. This evidence supports the course of treatment followed in our patient, who was initially started on IV PPI and later transitioned to oral therapy.

A study was conducted on 273 patients treated for caustic ingestion at Chang Gung Memorial Hospital in Taiwan between June 1999 and July 2006. All patients underwent endoscopy (EGD) within 24 hours of admission, and mucosal damage was assessed using Zargar's modified classification. Over a follow-up period of at least six months, the most common injury recorded was grade 3b (30.03%), followed by grade 2b (22.71%). The leading complication was stricture (24.18%), followed by aspiration pneumonia (11.36%) and respiratory failure (7.69%). Patients with grade 3b injuries faced significantly higher risks of prolonged hospital stays compared to those with grade 3a injuries [12]. While our patient’s condition remained stable during hospitalization, his loss to follow-up limits our understanding of any potential long-term complications [11].

Historically, endoscopy was the cornerstone of post-caustic ingestion management algorithms. However, its major limitation lies in its inability to reliably predict transmural necrosis, potentially leading to unnecessary surgery or delayed intervention, both of which can carry significant risks, including death. Implementing a CT-based algorithm for selecting patients for emergency surgery has significantly improved outcomes compared to endoscopy-based management. Recent studies demonstrate that contrast-enhanced CT surpasses endoscopy in detecting transmural gastrointestinal injuries following caustic ingestion and predicting esophageal stricture formation. Emergency endoscopy should be considered only under specific circumstances: (1) when CT is unavailable, (2) when CT with contrast is contraindicated (e.g., renal failure, iodine allergy), (3) when CT suggests transmural esophageal necrosis but interpretation is uncertain, or (4) in pediatric patients (grade 2A) [13].

Emergency surgery is essential for patients with signs of transmural necrosis in the gastrointestinal tract, aiming to remove all necrotic tissue. Laparotomy is the standard approach, with minimally invasive techniques used selectively in specialized centers. For combined esophageal and gastric injuries, esophagogastrostomy with cervical oesophagostomy is performed through both abdominal and cervical approaches. In cases of airway injury or significant mediastinal contamination, a transthoracic approach is preferred. However, for isolated gastric wall necrosis, total gastrectomy is recommended.

## Conclusions

This case highlights the deceptive nature of caustic ingestions, where mild initial symptoms can obscure serious internal injuries. It emphasizes the importance for rapid toxicologicalist and gastroenterologist intervention and ongoing follow-up to identify potential complications. Prompt evaluation and management are crucial, even when patients present with minimal symptoms, as small amounts of corrosive substances like hydrochloric acid can cause severe damage, such as gastric necrosis in this instance. Early endoscopy is vital for assessing the extent of injury and informing treatment decisions. Additionally, administering PPIs and closely monitoring for possible complications like peritonitis or necrosis are essential to preventing adverse outcomes. Finally, diligent follow-up is important to detect long-term sequelae, such as gastric strictures or other GI complications, which may develop even after apparent clinical recovery.
